# Negative learning bias is associated with risk aversion in a genetic animal model of depression

**DOI:** 10.3389/fnhum.2014.00001

**Published:** 2014-01-16

**Authors:** Steven J. Shabel, Ryan T. Murphy, Roberto Malinow

**Affiliations:** Section of Neurobiology, Department of Neuroscience and Division of Biology, Center for Neural Circuits and Behavior, University of California at San DiegoLa Jolla, CA, USA

**Keywords:** lateral habenula, reinforcement learning, depression, helplessness, cLH, risk aversion, reward, behavior

## Abstract

The lateral habenula (LHb) is activated by aversive stimuli and the omission of reward, inhibited by rewarding stimuli and is hyperactive in helpless rats—an animal model of depression. Here we test the hypothesis that congenital learned helpless (cLH) rats are more sensitive to decreases in reward size and/or less sensitive to increases in reward than wild-type (WT) control rats. Consistent with the hypothesis, we found that cLH rats were slower to switch preference between two responses after a small upshift in reward size on one of the responses but faster to switch their preference after a small downshift in reward size. cLH rats were also more risk-averse than WT rats—they chose a response delivering a constant amount of reward (“safe” response) more often than a response delivering a variable amount of reward (“risky” response) compared to WT rats. Interestingly, the level of bias toward negative events was associated with the rat's level of risk aversion when compared across individual rats. cLH rats also showed impaired appetitive Pavlovian conditioning but more accurate responding in a two-choice sensory discrimination task. These results are consistent with a negative learning bias and risk aversion in cLH rats, suggesting abnormal processing of rewarding and aversive events in the LHb of cLH rats.

## Introduction

The LHb, a key regulator of monoaminergic brain regions (Amat et al., 2001; Ji and Shepard, 2007; Hikosaka et al., 2008), is metabolically and synaptically hyperactive in helpless rats (Caldecott-Hazard, 1988; Shumake et al., 2003; Li et al., 2011, 2013). Furthermore, the LHb is activated by unexpected losses of reward and inhibited by unexpected gains of reward (Matsumoto and Hikosaka, 2007). Based on these findings, we hypothesized that helpless rats would be more sensitive to decreases in reward size and less sensitive to increases in reward size than control rats.

We tested this hypothesis by measuring how quickly cLH and WT rats switched their preference away from a response which was unexpectedly decreased in reward value (downshift test) or toward a response which was unexpectedly increased in reward value (upshift test) (Roesch et al., 2006). These tests allow one to measure sensitivity to decreases and increases in reward independently while controlling for differences in non-specific learning rates. We also measured risk-based choice in cLH and WT rats. We predicted that cLH rats would be more risk-averse than WT rats since either a greater sensitivity to decreases in reward value or a decreased sensitivity to increases in reward value would produce risk-averse behavior. Consistent with the hypothesis, we found that cLH rats were more sensitive to downshifts in reward size, less sensitive to upshifts in reward size, and more risk-averse than WT rats. cLH rats also showed slower appetitive Pavlovian conditioning yet more accurate choice in a two-choice sensory discrimination task. These results are consistent with a negative learning bias in cLH rats which predisposes them to risk-averse behavior.

## Results

cLH (*n* = 11) and WT (*n* = 13) rats were trained to press a centrally-located lever for presentation of light cues which directed them to the left or right for sucrose solution reward. There were three different light cues: a left light cue that signaled reward delivery on the left (forced-choice), a right light cue that signaled reward delivery on the right (forced-choice), and a double light cue (lights on left and right illuminated simultaneously) that signaled reward delivery on either side (free-choice). Rats were required to headpoke into the correct reward port during the forced-choice trials to initiate reward delivery, otherwise the trial was scored as an error and no reward was delivered on that trial. Forced-choice trials were used so the rats continued to sample both sides during manipulations of reward size, and free-choice trials were used to determine their preference for either the left or right sides (Roesch et al., 2006).

**Figure 1:**
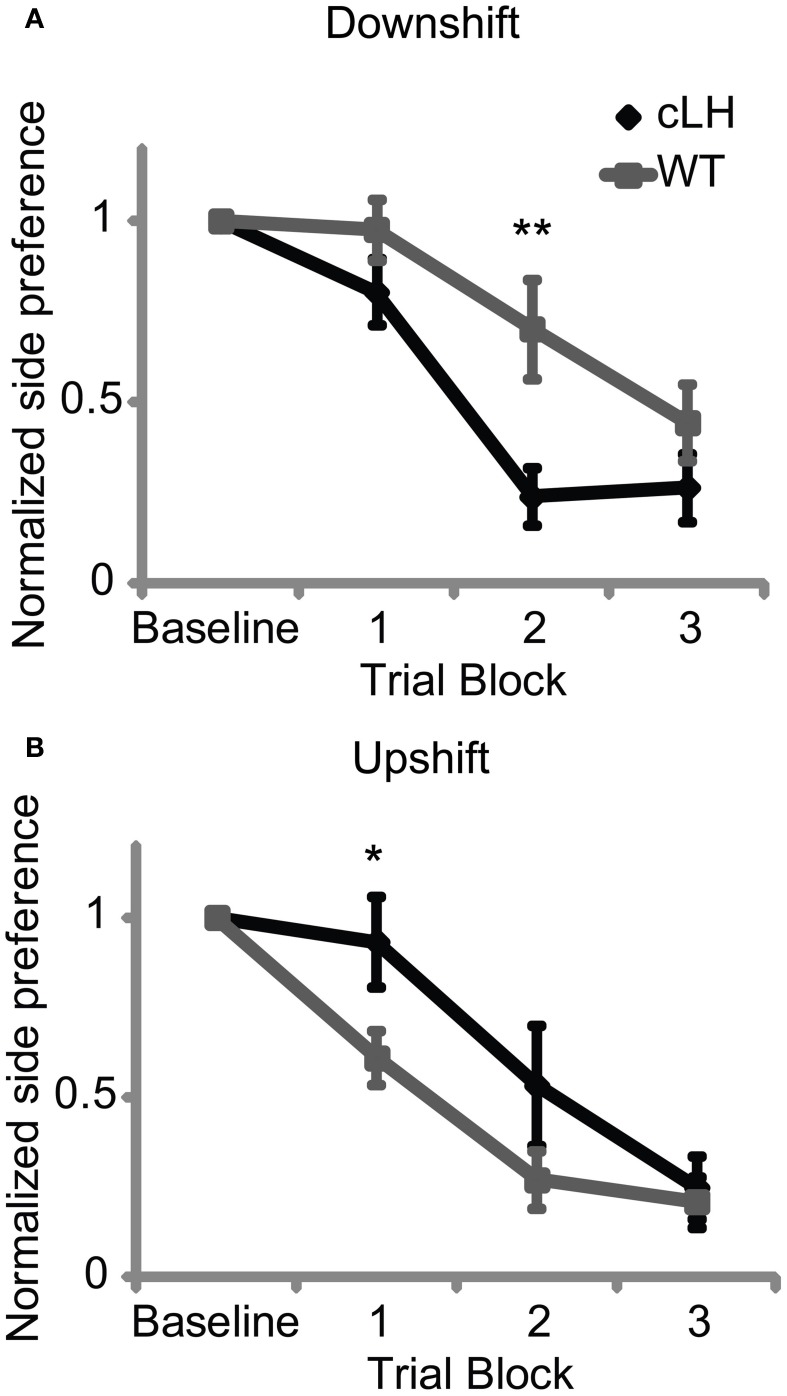
**Negative learning bias in cLH rats. (A)** cLH rats switched their preference away from the downshifted side more quickly than WT rats. **(B)** cLH rats switched their preference toward the upshifted side more slowly than WT rats. ^**^P < 0.01; ^*^P < 0.05. Student's *t*-tests on individual trial blocks. Error bars indicate s.e.m.

To measure rats' sensitivities to increases or decreases in reward size, rats were trained with an equal amount of reward on the left and right sides and then given upshift and downshift tests. During an upshift test, the size of the reward was increased on each rat's non-preferred side and the rate at which they changed their side preference during choice trials was used as a measure of their sensitivity to increases in reward size. During a downshift test, the size of the reward was decreased on each rat's preferred side and the rate at which they changed their side preference was used as a measure of their sensitivity to decreases in reward size. We first used large differences in reward size (two boluses of sucrose solution on side A vs. four boluses of sucrose solution on upshifted side B; 2 vs. 1 bolus for the downshift; see Materials and Methods) and found no difference in cLH and WT rats' sensitivities to the changes in reward size (ANOVA: all *F*s < 3, *P* > 0.05, data not shown; see Materials and Methods for description of statistical analysis). However, when using smaller changes in reward size (three vs. four boluses for the upshift; three vs. two boluses for the downshift), cLH rats were faster to switch their preference during the downshift (Figures 1A, S1) but slower to shift their preference during the upshift (Figures 1B, S1; ANOVA: interaction between rat group and shift type, *F*_(1, 23)_ = 17.8, *P* < 0.0001; 3-way interaction between rat group, shift type, and trial block, *F*_(3, 69)_ = 3.5, *P* = 0.02). After each of the downshift and upshift sessions, rats were given another session to test their ability to discriminate the sizes of the rewards used during the shifts. There was no difference between cLH and WT rats' abilities to discriminate the sizes of the rewards used during the downshift (preference for side delivering more reward: cLH, 78 ± 5%, WT, 73 ± 4%, *P* = 0.44, Student's *t*-test) or the upshift (preference for side delivering more reward: cLH, 69 ± 5%, WT, 75 ± 3%, *P* = 0.28, Student's *t*-test), suggesting that cLH and WT rats have similar abilities to discriminate reward magnitudes in these conditions. There was also no difference between the groups in baseline preference for the downshifted or upshifted side during the downshift or upshift sessions (baseline preference during downshift: cLH, 60 ± 10%, WT, 56 ± 6%, *P* = 0.68; upshift: cLH, 85 ± 7%, WT, 87 ± 3%; *P* = 0.74; Student's *t*-tests). These results suggest that cLH rats have a negative learning bias—they are more sensitive to decreases in reward but less sensitive to increases in reward than WT rats.

**Figure 2:**
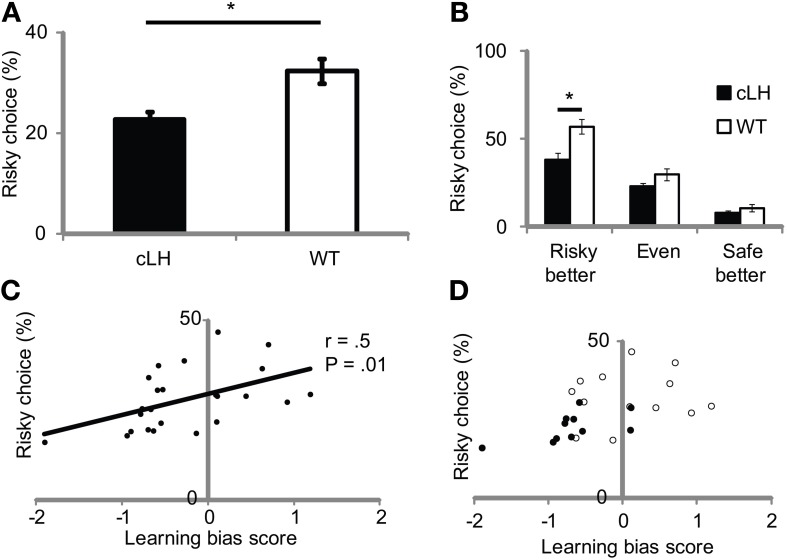
**Risk-averse choice in cLH rats and its relationship to negative learning bias. (A)** cLH rats chose the variable/risky side less than WT rats (averages of variable/risky better, even, and constant/safe better conditions used for comparison between groups). **(B)** cLH rats chose the variable/risky side less than WT rats in the variable/risky better condition, when risk was greatest. **(C)** Learning bias is correlated with risky choice. **(D)** Same as **(C)** with symbols denoting cLH (filled circles) and WT (open circles) groups. ^*^P < 0.01. Student's *t*-test. Error bars indicate s.e.m.

To test if the negative learning bias in cLH rats led to risk-aversion, we measured rats' preferences for either a “risky,” variable-sized reward option (one or seven boluses) or a “safe,” constant-sized reward option (two boluses) on free-choice trials. If cLH rats are risk-averse, they should choose the variable/risky option less than WT rats. We measured variable/risky choice under three conditions—when the variable/risky option delivered more reward on average than the constant/safe option, the same amount of reward, or less reward on average than the constant/safe option. These conditions not only vary in the amount of expected reward on the variable/risky side but also in the amount of risk associated with the variable/risky option (risky better > even > safe better), where risk is defined as the variance of the reward size (Markowitz, 1952). Consistent with the hypothesis, cLH rats chose the variable/risky side less than WT rats [Figure 2A; ANOVA: main effect of rat group, *F*_(1, 23)_ = 10.0, *P* = 0.002; main effect of risk condition, *F*_(2, 46)_ = 48.1, *P* < 0.0001; interaction between rat group and risk condition, *F*_(2, 46)_ = 2.5, *P* = 0.09]. cLH rats modulated their decision-making in accordance with the expected reward on the variable/risky side, suggesting that cLH rats are able to calculate expected reward normally and use this information to guide their decision-making. We note that the difference in risky choice between cLH and WT rats only reached statistical significance in the “risky better” condition—the condition with the most risk associated with the variable/risky option (Figure 2B).

To determine if a negative learning bias predicted risk-averse behavior, we first computed a learning bias score from the rate at which rats changed their preference in the upshift and downshift tests (see Materials and Methods). If a rat changed its preference faster after an upshift than downshift, its learning bias score would be positive. If it changed its preference slower after an upshift than downshift, its learning bias score would be negative. We then computed the correlation between the rats' learning bias scores and their average risky choice scores and found a significant positive relationship between learning bias and risky choice (Figures 2C,D; *r* = 0.5, *P* = 0.01, *n* = 24 rats; *r* = 0.59, *P* = 0.053, *n* = 11 cLH rats). This relationship is consistent with the idea that a negative learning bias in cLH rats contributes to their risk-averse behavior.

**Figure 3:**
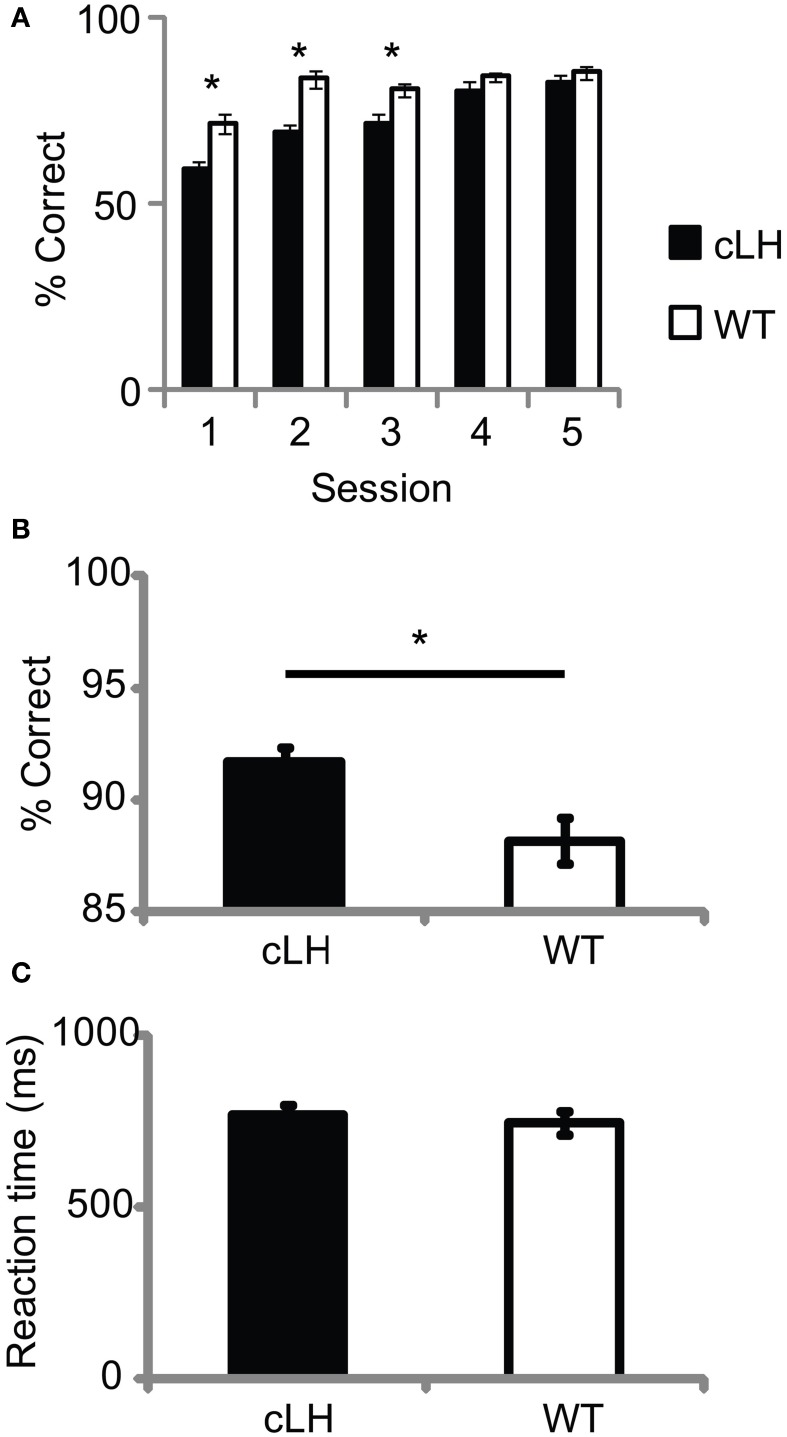
**Pavlovian conditioning and forced-choice accuracy. (A)** Slower Pavlovian conditioning in cLH rats. **(B)** cLH rats responded more accurately during forced-choice trials with even, constant reward sizes on both sides. **(C)** Reaction time from lever press to sucrose delivery port during same forced-choice trials as in **(B)**. ^*^P < 0.01. Student's *t*-test on individual sessions in **(A)**. Error bars indicate s.e.m.

We found other differences between cLH and WT rats that were consistent with abnormal reward processing in cLH rats. cLH rats showed slower Pavlovian conditioning during initial light-reward pairings [before rats were trained to press a lever for light presentation; Figure 3A; ANOVA: main effect of rat group, *F*_(1, 23)_ = 43.5, *P* < 0.0001; main effect of session number, *F*_(4, 92)_ = 25.7, *P* < 0.0001; interaction between rat group and session number, *F*_(4, 92)_ = 3.3, *P* = 0.01], however, once lever training was complete, cLH rats were more accurate on forced-choice trials (when they had to respond to one of the lights after a lever press; Figure 3B) with even, constant reward sizes on both sides. This was not due to more motor impulsivity in WT rats since there was no difference in reaction times between lever press and entry into the reward receptacles between the two groups (Figure 3C) and WT rats were actually slower to respond to the insertion of the lever than cLH rats (cLH, 0.58 ±.03 s; WT, 0.73 ±.06 s; *P* < 0.05). Forced-choice accuracy was correlated with a negative learning bias (*r* = 0.49, *P* = 0.01, *n* = 24 rats), but Pavlovian conditioning was not (average Pavlovian conditioning accuracy for first three sessions vs. negative learning bias, r = −0.34, *P* = 0.11, *n* = 24 rats).

## Discussion

Here we show that cLH rats have a negative learning bias when responding to small changes in reward size and this negative learning bias is associated with risk aversion. We also found that cLH rats show impaired appetitive Pavlovian conditioning yet more accurate forced-choice responses after a lever press.

The lesser sensitivity to an increase in reward size in cLH rats is consistent with impaired appetitive Pavlovian conditioning and other studies that found altered sucrose consumption (Sanchis-Segura et al., 2005; Shumake et al., 2005) and less operant responding for reward in a progressive ratio test (Vollmayr et al., 2004). Together, these findings indicate that cLH rats are less sensitive to reward than control rats. Less responding during a progressive ratio test is also consistent with our finding that cLH rats are more sensitive to decreases in reward size, since progressive ratio tests measure operant responding after repeated omissions of reward.

Our results are also consistent with a study that found a negative bias in cLH rats' interpretation of ambiguous cues (Enkel et al., 2010a). In this study, rats were trained to press a lever to avoid shock in response to one sound and to press another lever to get reward in response to another sound. During the test, rats were given sounds that were perceptually between the two trained sounds (i.e., ambiguous cues). cLH rats chose the negative, shock-avoidance lever more often than control rats during presentation of the ambiguous cues. Notably, this result and our finding of greater risk aversion in cLH rats are consistent with a pessimistic bias in cLH rats, since both behaviors can be explained by a greater tendency to expect a negative outcome. Given the association we found between risk aversion and learning bias, we hypothesize that cLH rats choose pessimistically and this is due to excessive learning from punishments and impaired positive reinforcement learning, perhaps because of altered processing of punishments and rewards in the LHb.

Not only were cLH rats faster to shift their responses toward the bigger reward during the downshift sessions, they were also more accurate than WT rats on the forced-choice trials. These two observations might be causally related since there was a positive relationship between forced-choice accuracy and negative learning bias. Accordingly, since errors also involve unexpected decreases (omission) of reward, cLH rats may be more sensitive to errors and learn more from them. We note that learning from errors is particularly instructive once an animal understands the constraints of the task, but not as informative during the initial stages of learning, when exploration is needed to determine which behaviors will be rewarded. This may be why cLH rats were not more accurate (in fact, they were less accurate) during Pavlovian conditioning, when rewards were more instructive than errors. cLH rats may have been less accurate during Pavlovian conditioning because they are less responsive to reward (Vollmayr et al., 2004; Sanchis-Segura et al., 2005; Shumake et al., 2005), although we note that we found no significant relationship between positive learning bias and accuracy during Pavlovian conditioning.

Importantly, differences between cLH and WT rats were found on the downshift and upshift tests only when we used small changes in reward size. When big changes in reward size were used, there was no difference in the rate at which the groups changed their response preference. Possibly, this is because the big changes in reward size were too obvious and recruited explicit memory systems (such as the hippocampus) that may function similarly in cLH and WT rats, masking differences in implicit reward memory function (governed by the basal ganglia). We also note that congenitally non-helpless rats were not tested in our experiments. It would be interesting to determine if differences in their behavior are opposite to those of cLH rats on the tests reported here.

Although cLH rats were bred for learned helplessness, there are many differences in behavior between cLH and WT or non-helpless rats besides helplessness—sucrose consumption (Sanchis-Segura et al., 2005; Shumake et al., 2005), operant responding for reward (Vollmayr et al., 2004), reaction to stress (King et al., 1993, 2001; Enkel et al., 2010b), fear conditioning and extinction (Shumake et al., 2005), response to novelty (Shumake et al., 2005), negative ambiguous-cue interpretation (Enkel et al., 2010a), and now negative learning bias, risk aversion, appetitive Pavlovian conditioning, and response accuracy. This suggests that disposition to helpless behavior is associated with several other differences in behavior, many of which are similar to depressive symptoms (Hasler et al., 2004; Roiser et al., 2012). It remains unclear why breeding rats for helplessness would produce rats with these other differences in behavior. One possibility is that greater sensitivity to negative events (i.e., punishment) in some rats predisposes them to be more sensitive to the shock that induces helplessness, while lesser sensitivity to rewarding events predisposes them to be less sensitive to the termination of shock during helplessness testing. This negative learning bias may also explain many of the other behaviors listed above. A better understanding of the core neurobiological mechanisms underlying the behavior of cLH rats will help shed light on the precise nature of the behavioral differences seen in cLH rats and perhaps the etiology of depression. Given that cLH rats have altered processing of increases and decreases in reward size and lateral habenula hyperactivity, it will be especially interesting to determine how the LHb of cLH rats processes increases and decreases in reward size.

## Materials and methods

### Animals

Male, adult, age-matched cLH and WT Sprague-Dawley (Harlan) rats were used. Rats were singly-housed and kept on a 12/12 h light-dark cycle (lights on/off at 6 am/6 pm). Rats were water restricted and given access to water for 90 min/per day, following the end of each training or test session. All procedures involving animals were approved by the Institute Animal Care and Use Committees of the University of California, San Diego.

### Equipment

All training and testing was done in standard rat operant chambers (Med Associates Inc.). A houselight provided constant, low-level illumination. Recessed sucrose delivery ports with infrared beams that detected head entry were located on the left and right sides of the chamber. Two lights, used as conditioned and discriminative stimuli were located a few centimeters above the ports, one light above each port. One retractable lever was located between the ports. Sucrose solution was delivered to a well in each port via a programmable pump.

### Behavioral procedures

First, rats were given five sessions of Pavlovian conditioning that lasted 60 min each. During the first two sessions, lights located over the left and right sucrose delivery ports were illuminated randomly (one at a time), 15 s after the start of the last trial, with the exception that the delivery of sucrose must have been consumed before the next light was illuminated. Two boluses of 20 μ l of 10% sucrose solution were delivered concurrent with light illumination in the port underneath the illuminated light. No further lights or sucrose were delivered until after the rat entered the correct port for > 500 ms (i.e., drank the sucrose solution). If the rat entered the wrong port after light illumination, the trial was scored an error but the rat could still get sucrose solution by subsequently entering the correct port. The cue light was only turned off once the rat responded to the correct port. The final 3 Pavlovian conditioning sessions were similar to the first 2 except that sucrose delivery was contingent on the rat responding to the correct port and therefore sucrose delivery started only after the rat entered the correct port. If the rat responded to the wrong port, the light was turned off, no sucrose was delivered, and the trial was scored an error.

After five sessions of Pavlovian conditioning, rats were trained to press a lever for illumination of one of the cue lights. If they responded to the correct port after light illumination, two boluses of sucrose solution were delivered, as in the last 3 Pavlovian conditioning sessions. During initial training, the lever was not retracted after a lever press, but after the rat began to press the lever, it was given another session in which the lever was retracted after a lever press and reinserted 6 s after sucrose consumption. After a rat pressed the lever at least 100 times in 60 min, it started baseline lever-pressing sessions. During six sessions of baseline lever-pressing, rats pressed the lever and randomly received one of three cue lights: left light (forced-choice) which required a response to the left reward port, right light (forced-choice) which required a response to the right reward port, or both lights (free-choice) after which they could respond to either side to get reward. Responses to the wrong side during forced-choice trials were scored an error and resulted in light off and no sucrose delivery for that trial. These sessions lasted 60 min and were used to determine rats' accuracy and reaction time during forced-choice trials (Figures 3B,C). All subsequent sessions were similar to these sessions, but varied in the amount of reward given on each side.

After six sessions of baseline lever-pressing, rats were given an upshift session with a large increase in reward size. During these sessions, baseline preference for the left and right sides was determined during the first 21 free-choice trials. Reward size was then increased from two to four boluses on each rat's non-preferred side, while reward size remained at two boluses on the preferred side. The session ended after 180 total trials (60 free-choice, 60 forced-left, and 60 forced-right). The same reward sizes (4 and 2) were used for the entirety of the following session (similar to the end of the upshift test). Next, the rats were given a downshift session with a large decrease in reward size. During the baseline period which ended after 21 free-choice trials, rats continued with four and two boluses of reward. After the baseline period, the side which delivered four boluses was downshifted to one bolus of reward. The session ended after 180 total trials.

After the first large upshift and downshift tests, rats were trained for two sessions with three smaller boluses (~15 μ l each) of sucrose solution on each side. Next, rats were given a small downshift session in which the side opposite to the one manipulated during the large shift sessions was downshifted from three boluses to two boluses of reward after a baseline period that ended after 21 free-choice trials. The session ended after 240 total trials (80 free-choice, 80 forced-left, and 80 forced-right). We used more trials during the smaller shifts than the larger shifts because we anticipated that rats would be slower to shift their preference after a small change in reward size. After one further session with three boluses of reward on one side and two boluses of reward on the other to measure reward magnitude discrimination, rats were given a small upshift session. This session was similar to previous shift sessions except that the side delivering two boluses of reward was upshifted to four boluses of reward after the baseline period. The following day, rats were given another session with the same difference in reward size as was used during the upshift session—three and four boluses of reward, delivered on the same sides as during the upshift session—to measure reward magnitude discrimination.

Before risk aversion training, rats were trained with two boluses of reward as used initially. During risk aversion training, one side was randomly designated the risky side and the other side was the safe side, which always delivered two boluses of reward. Half the cLH and WT rats started with the “risky better” condition in which the risky side produced one bolus of reward 75% of the time and seven boluses of reward 25% of the time on average. The other half started with the “safe better” condition in which the risky side produced one bolus of reward 90% of the time and seven boluses 10% of the time. Rats were given 3 risk aversion training sessions, followed by two test sessions which were identical to the training sessions. Next, the risky and safe sides were switched for each rat and they were given three more training sessions, followed by two test sessions. Risky choice scores during the free-choice trials from the four test sessions were averaged together for each rat to produce a single score for that condition (risky better or safe better). Next, the risky and safe sides were switched again and the rats were given five total sessions (three training, two test as before) in the “even” condition, in which the risky side delivered one bolus 5/6 of the time and seven boluses 1/6 of the time. The risky and safe sides were switched again and rats were given five more sessions as before. Finally, rats were tested in their last remaining condition (either risky better or safe better) as described previously.

### Data analysis

Side preference was normalized for each rat by dividing side preference during each trial block by side preference during the baseline block. The baseline block consisted of 21 free-choice trials, blocks 1 and 2 consisted of 20 free-choice trials, and block 3 consisted of 19 free-choice trials.

Learning bias was defined as:

ΔU – ΔD

Where ΔU = the change in preference during the upshift (during block 1)

And ΔD = the change in preference during the downshift (during block 2)

For Figure S1, side preference was computed for each rat as a running average of the side chosen on the indicated trial and 5 trials before and after the indicated trial (11 trials total).

### Statistical analysis

Student's *t*-tests were used with *P* < 0.05 deemed significant. For analysis of the upshifts and downshifts, we performed an analysis of variance with 3 factors—rat group (cLH or WT), shift type (downshift or upshift), and trial block (baseline, block 1, block 2, block 3)—followed by Student's *t*-tests. For analysis of the risk aversion tests, we performed an analysis of variance with 2 factors—rat group (cLH or WT) and risk condition (risky better, even, and safe better)—followed by Student's *t*-tests. For analysis of Pavlovian conditioning, we performed an analysis of variance with 2 factors—rat group (cLH or WT) and session number (1,2,3,4,5)—followed by Student's *t*-tests.

## Author contributions

Steven J. Shabel, Ryan T. Murphy, and Roberto Malinow designed the experiments. Steven J. Shabel and Ryan T. Murphy performed the experiments. Steven J. Shabel and Roberto Malinow analyzed the data. Steven J. Shabel and Roberto Malinow wrote the manuscript.

### Conflict of interest statement

The authors declare that the research was conducted in the absence of any commercial or financial relationships that could be construed as a potential conflict of interest.

## Supplementary material

Figure S1 **Behavior during downshift and upshift sessions (same data as shown in Figure 1, but values have not been normalized to baseline period). (A)** Choice of initially preferred side during downshift test in cLH and WT rats. **(B)** Choice of initially preferred side during upshift test in cLH and WT rats.
