# Supplementary figures and images for: Negative learning bias is associated with risk aversion in a genetic animal model of depression
Source: Front Hum Neurosci. 2014 Jan 16;8:1. doi: 10.3389/fnhum.2014.00001 (PMC3893716; doi:10.3389/fnhum.2014.00001)

A

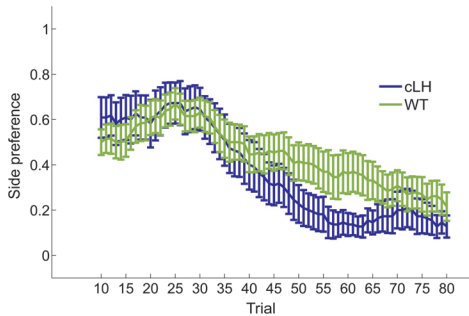

B

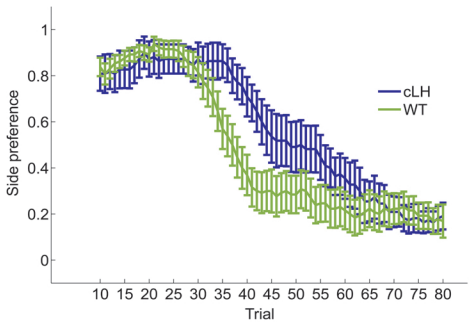

Supplement: Figure S1 — Behavior during downshift and upshift sessions (same data as shown in Figure 1, but values have not been normalized to baseline period). (A) Choice of initially preferred side during downshift test in cLH and WT rats. (B) Choice of initially preferred side during upshift test in cLH and WT rats. [file Presentation1.PDF]
